# A surge in endogenous spermidine is essential for rapamycin-induced autophagy and longevity

**DOI:** 10.1080/15548627.2024.2396793

**Published:** 2024-08-30

**Authors:** Sebastian J. Hofer, Ioanna Daskalaki, Mahmoud Abdellatif, Ulrich Stelzl, Simon Sedej, Nektarios Tavernarakis, Guido Kroemer, Frank Madeo

**Affiliations:** a Institute of Molecular Biosciences, NAWI Graz, University of Graz, Graz, Austria; b BioTechMed-Graz, Graz, Austria; c Field of Excellence BioHealth, University of Graz, Graz, Austria; d Institute of Molecular Biology and Biotechnology, Foundation for Research and Technology – Hellas, Heraklion, Greece; e Department of Biology, School of Sciences and Engineering, University of Crete, Heraklion, Greece; f Division of Cardiology, Medical University of Graz, Graz, Austria; g Institute of Pharmaceutical Sciences, Pharmaceutical Chemistry, University of Graz, Graz, Austria; h Institute of Physiology, Faculty of Medicine, University of Maribor, Maribor, Slovenia; i Division of Basic Sciences, School of Medicine, University of Crete, Heraklion, Greece; j Centre de Recherche des Cordeliers, Équipe Labellisée par la Ligue Contre le Cancer, Université de Paris Cité, Sorbonne Université, Inserm U1138, Institut Universitaire de France, Paris, France; k Metabolomics and Cell Biology Platforms, Gustave Roussy Cancer Center, Université Paris Saclay, Villejuif, France; l Institut du Cancer Paris CARPEM, Department of Biology, Hôpital Européen Georges Pompidou, AP-HP, Paris, France

**Keywords:** Aging, autophagy, lifespan, rapamycin, spermidine, MTOR

## Abstract

Acute nutrient deprivation (fasting) causes an immediate increase in spermidine biosynthesis in yeast, flies, mice and humans, as corroborated in four independent clinical studies. This fasting-induced surge in spermidine constitutes the critical first step of a phylogenetically conserved biochemical cascade that leads to spermidine-dependent hypusination of EIF5A (eukaryotic translation initiation factor 5A), which favors the translation of the pro-macroautophagic/autophagic TFEB (transcription factor EB), and hence an increase in autophagic flux. We observed that genetic or pharmacological inhibition of the spermidine increase by inhibition of ODC1 (ornithine decarboxylase 1) prevents the pro-autophagic and antiaging effects of fasting in yeast, nematodes, flies and mice. Moreover, knockout or knockdown of the enzymes required for EIF5A hypusination abolish fasting-mediated autophagy enhancement and longevity extension in these organisms. Of note, autophagy and longevity induced by rapamycin obey the same rule, meaning that they are tied to an increase in spermidine synthesis. These findings indicate that spermidine is not only a “caloric restriction mimetic” in the sense that its supplementation mimics the beneficial effects of nutrient deprivation on organismal health but that it is also an obligatory downstream effector of the antiaging effects of fasting and rapamycin.

**Abbreviation**: EIF5A: eukaryotic translation initiation factor 5A; IGF1: insulin like growth factor 1; MTOR: mechanistic target of rapamycin kinase; ODC1: ornithine decarboxylase 1; TFEB: transcription factor EB.

Polyamines, including putrescine, spermidine, and spermine, as well as their precursors and regulatory enzymes, are highly conserved across species. Our previous work has highlighted the multifaceted consequences of spermidine supplementation, which exerts cardio- and neuroprotective effects, stimulates autophagy and mitochondrial function, and extends lifespan in a variety of laboratory models. These findings are particularly salient given that polyamine metabolism, predominantly regulated by the pacemaker enzyme ODC1 (ornithine decarboxylase 1), is a critical driver of cellular growth. The concordant activity of polyamines, stimulation of cell growth and induction of autophagy, differs from the discordant action of MTOR (mechanistic target of rapamycin kinase), which stimulates cell growth but represses autophagy.

Rapamycin, a potent and selective inhibitor of MTOR, has long been recognized for its ability to extend longevity across species, including yeast and worms. Our recent data demonstrate that rapamycin treatment in yeast is accompanied by a concomitant increase in endogenous spermidine levels [1]. Notably, the inhibition of endogenous spermidine synthesis significantly attenuates the autophagy-inducing and longevity-promoting effects of rapamycin in yeast, human cell lines and worms, underscoring the essential role of polyamine metabolism in these processes. Accordingly, our study provides further compelling evidence that the pro-autophagic and lifespan-extending effects of dietary restriction and intermittent fasting – physiological triggers that shut down TOR signaling – are largely dependent on functional endogenous polyamine metabolism (Figure 1). Acute fasting is associated with an increase in polyamine levels across multiple species and tissues, supporting our hypothesis that this rise in polyamines is necessary to trigger the autophagic cascade. Moreover, genetic perturbation of MTOR activity in transgenic mice further corroborates our findings, as changes in spermidine levels align with expected autophagic outcomes. Notably, our previous work has shown that spermidine can effectively counteract the downstream effects of hyperactive INS (insulin)-IGF1 (insulin like growth factor 1) signaling during cardiac aging in mice.

**Figure 1.**
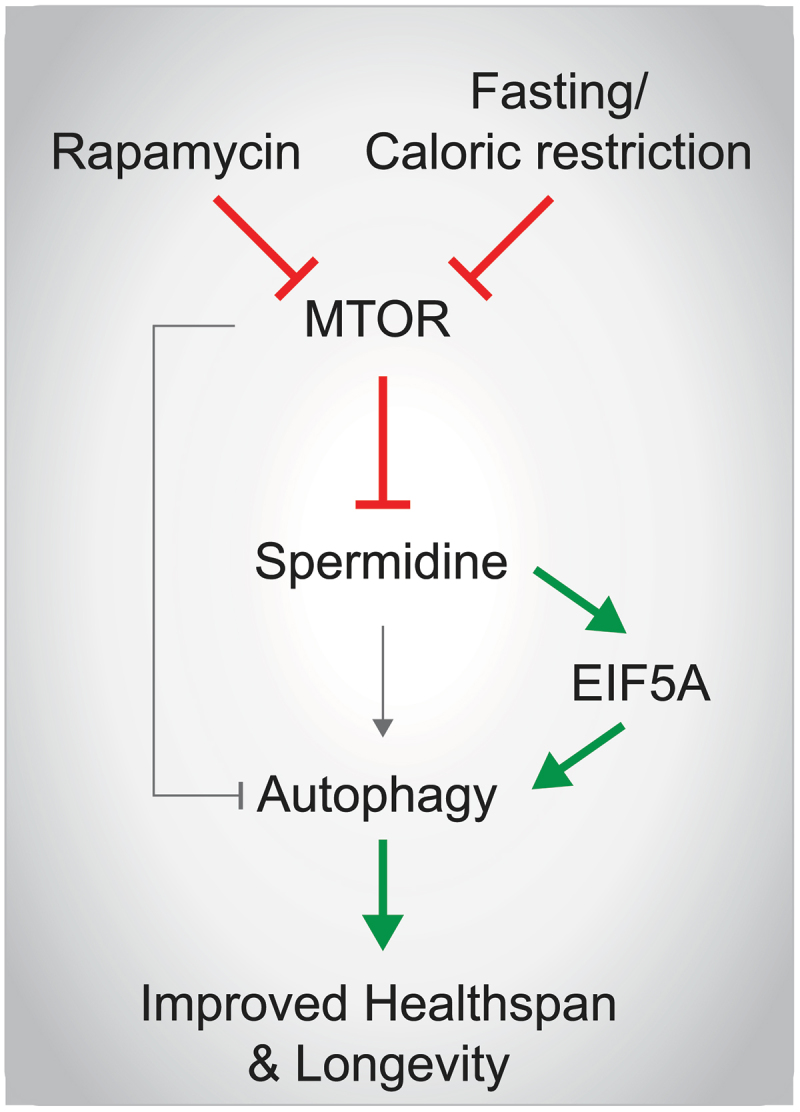
MTOR and the polyamine pathway during nutrient deprivation. In our recent work, we described a novel mechanism by which fasting and caloric restriction lead to the induction of polyamine metabolism and the increased activation of the translation factor EIF5A via the spermidine-dependent post-translational hypusination modification. Activated EIF5A is required for the full autophagy-inducing and longevity-promoting properties of fasting. MTOR functions as a negative regulator of spermidine synthesis and autophagy in our settings. Consequently, pharmacologically inhibiting MTOR with rapamycin also elicits spermidine-dependent effects on autophagic flux and lifespan regulation.

In yeast, proteomic analyses reveal that TOR complex 1- and TOR complex 2-associated factors exhibit altered responses to starvation when endogenous polyamine synthesis is disrupted. Specifically, the loss of polyamine synthesis results in a delayed deactivation of early TOR activity during nitrogen starvation in *odc1* knockout strains – a defect that can be rescued by polyamine supplementation. This suggests a potential checkpoint mechanism to fine-tune autophagy regulation. Interestingly, excess polyamines do not influence TOR activity in wild-type cells, indicating a tightly regulated interplay between these pathways under nutrient-rich conditions. The precise mechanisms through which polyamines affect MTOR signaling, and vice versa, remain incompletely understood and likely operate on multiple levels. In the short term, polyamine-controlled translational events are unlikely to explain altered MTOR signaling, given the minute scale of events. At later stages such spermidine-modulated, EIF5A hypusination-dependent translational processes, may modulate MTOR signaling. Additionally, polyamines might exert their influence on MTOR components or signaling factors indirectly through metabolic alterations. Because MTOR senses amino acid concentrations, including those of arginine and methionine, shifts in such amino acids, as they are found in polyamine-deficient cells, could contribute to the dysregulated MTOR activity.

Furthermore, we observed that blocking endogenous polyamine synthesis diminishes rapamycin-induced autophagy in yeast, human cell lines, and *C. elegans*. This finding suggests that the well-documented lifespan-extending effects of rapamycin are, at least in part, contingent upon the polyamine pathway. This hypothesis has been corroborated in yeast and nematodes. Thus, inhibition of ODC1 prevents rapamycin-induced lifespan extension in these model organisms.

Importantly, spermidine supplementation enhances immune responses in aged mice, improving tumor cell surveillance and vaccine efficacy. While rapamycin is one of the first pharmacological interventions shown to extend the lifespan of mice, concerns about immunosuppressive side effects of rapamycin have been raised. Indeed, the first clinical indication for rapamycin was immunosuppression in the context of organ transplantation. It is tempting to speculate, yet remains to be demonstrated, that such immunosuppressive effects can be mitigated by co-administering rapamycin with spermidine, by using rapamycin in a discontinuous fashion, or by a combination of both approaches.

In conclusion, our research uncovers a reciprocal dependency between MTOR signaling and polyamine metabolism, adding a new layer of complexity to the regulation of autophagy in response to nutrient availability. We speculate that the cross-regulation of MTOR and polyamine pathways evolved as a mechanism to fine-tune cellular growth and ensure cellular survival under varying environmental conditions. Future investigations should aim at elucidating the interplay between these pathways across different cell types and stages of the lifespan. This knowledge could aid in therapeutically exploiting the MTOR-polyamine axis. The strategic combination of rapamycin with spermidine in a sophisticated rhythmic fashion holds promise as a novel approach to maximizing therapeutic efficacy while minimizing adverse effects. We hypothesize that the negative side effects of continuous rapamycin treatment could be effectively counterbalanced by supplementary spermidine administration, paving the way for ever more efficient geroprotective interventions.

In sum, we propose that the MTOR and polyamine pathways function within a finely tuned regulatory network that orchestrates cell growth in response to nutrient availability and metabolic demands. MTOR inhibition upregulates polyamine synthesis, possibly to maintain essential cellular functions, including autophagy, and to prevent compromised cellular growth. We surmise that a detailed mechanistic exploration of this network may yield further clues for antiaging therapies.
